# Mesoporous Titania Powders: The Role of Precursors, Ligand Addition and Calcination Rate on Their Morphology, Crystalline Structure and Photocatalytic Activity

**DOI:** 10.3390/nano4030583

**Published:** 2014-07-30

**Authors:** Elisabetta Masolo, Manuela Meloni, Sebastiano Garroni, Gabriele Mulas, Stefano Enzo, Maria Dolors Baró, Emma Rossinyol, Agnieszka Rzeszutek, Iris Herrmann-Geppert, Maria Pilo

**Affiliations:** 1 Department of Chemistry and Pharmacy, University di Sassari and INSTM, Via Vienna 2, I-07100 Sassari, Italy; E-Mails: manu-m-89@hotmail.it (M.M.); sgarroni@uniss.it (S.G.); mulas@uniss.it (G.M.); enzo@uniss.it (S.E.); mpilo@uniss.it (M.P.); 2 Departament de Física, Universitat Autònoma de Barcelona, E-08193 Bellaterra, Spain; E-Mail: dolors.baro@uab.es; 3 Servei de Microscòpia, Universitat Autònoma de Barcelona, E-08193 Bellaterra, Spain; E-Mail: Emma.Rossinyol@uab.cat; 4 Helmholtz Centre Geesthacht, Institute for Materials Research, Max-Planck-Str. 1, 21502 Geesthacht, Germany; E-Mails: agnieszka.rzeszutek@hzg.de (A.R.); iris.herrmann-geppert@hzg.de (I.H.-G.); 5 Helmut-Schmidt-University, Functional Materials, Holstenhofweg 85, 22043 Hamburg, Germany

**Keywords:** titania, EISA method, powders, photocatalysis, water splitting

## Abstract

We evaluate the influence of the use of different titania precursors, calcination rate, and ligand addition on the morphology, texture and phase content of synthesized mesoporous titania samples, parameters which, in turn, can play a key role in titania photocatalytic performances. The powders, obtained through the evaporation-induced self-assembly method, are characterized by means of *ex situ* X-Ray Powder Diffraction (XRPD) measurements, N_2_ physisorption isotherms and transmission electron microscopy. The precursors are selected basing on two different approaches: the acid-base pair, using TiCl_4_ and Ti(OBu)_4_, and a more classic route with Ti(O^i^Pr)_4_ and HCl. For both precursors, different specimens were prepared by resorting to different calcination rates and with and without the addition of acetylacetone, that creates coordinated species with lower hydrolysis rates, and with different calcination rates. Each sample was employed as photoanode and tested in the water splitting reaction by recording I-V curves and comparing the results with commercial P25 powders. The complex data framework suggests that a narrow pore size distribution, due to the use of acetylacetone, plays a major role in the photoactivity, leading to a current density value higher than that of P25.

## 1. Introduction

Titania is one of the most studied materials because of its unique properties that include chemical stability, low cost, nontoxicity together with optimal electronic, and optical capacity, that make it a promising candidate for photocatalytic applications [1,2,3,4,5,6,7]. Since the discovery of photoelectrocatalytic hydrogen production from the splitting of water by Fujishima and Honda [8], titania has been the subject of an intense scientific research because of its wide band gap (3.2 eV) that ensures both hydrogen and oxygen production without providing any further overpotentials [9]. As a catalyst, usually a large surface area and a nanocrystalline titania is highly desirable [10,11]. In this frame, ordered mesoporous materials show very large surface areas and well-arranged pore channels that ensure great mass diffusion within frameworks [12]. Among the wide variety of synthetic strategies used to obtain mesoporous titania, the most promising method seems to be the evaporation-induced self-assembly (EISA) [13]: starting from a diluted solution of a surfactant and the addition of a transition metal oxide precursors, by slow evaporation of the solvent under controlled moisture level, it is possible to synchronize the formation of micelles from the surfactant and the condensation of the inorganic species giving rise to a highly-ordered mesostructured hybrids. There are several factors that have to be taken into account during the EISA process: (I) most titania precursors have fast hydration kinetics thus tending to react before the formation of micelles (II) chemical composition and phase structure of titania are difficult to control and the synthesis is very sensitive to aging and calcination conditions [14]. In order to slow down the reaction kinetics of titania precursors, usually alkoxides, the initial solution is kept at low pH levels by adding hydrocloric acid, implying the addition of water to the organic solvent and thus leading to uncontrolled processes. A valid alternative to HCl is represented by the addtion of titanium tetrachloride together with a titanium alkoxide in a so-called acid-base pairs approach [15,16], with the former being the pH “adjustor” and hydrolysis-condensation controller. Another suitable method of controlling the high reactivity of transition metals is the addition of coordinating molecules like acetylacetone that chelate the precursor forming metal complexes with lower hydrolysis kinetics [17]. Most of the reported work on titania obtained through EISA method concerns the preparation of well ordered thin films [18,19] and only few works can be found concerning the preparation of ordered mesoporous and crystalline massive powders [14,16], that can be more flexible for practical use.

In this paper, we report the synthesis of mesoporous crystalline titania powders by the EISA method according to the acid-base pairs concept by using TiCl_4_ and Ti(OBu)_4_ as precursors or a more “classic” route by adding HCl to titanium isopropoxide. The main intent is to clarify the influence of a coordinating ligand as the acetylacetone on the samples morphology and crystalline structure while varying precursors and calcination rates. Furthermore, in order to find clear evidence on the effect of these parameters on the photocatalytic activity of the powders, all titania samples were tested as photoanodes in a photoelectrochemical cell (PEC) for the water splitting reaction and their performances were compared to the corresponding data of P25 powders, considered as a reference material.

## 2. Results and Discussion

A total of eight samples were synthesized by following the procedure reported in the experimental section (Table 1). The intent was to tentatively relate the addition of coordinating ligands like acetylacetone, the different calcination rates and different precursors to the final morphology and crystal structure of our titania powders, keeping constant the amount of titania precursors and the pH level in all the synthesis. The calcination temperature of 673 K was chosen taking into account the temperature needed for the subsequent preparation of the titania electrodes and 12 h of calcination were necessary for the complete removal of the surfactant P123. P25 was chosen for comparison for evaluating the electrochemical performances of the powders.

**Table 1:** Synthetic parameters. Used in the preparation of mesoporous titania samples.

Samples	Precursors	Acetylacetone (mL)	Heating Rate (K∙min^−1^)
1	TiCl_4_—Ti(OBu)_4_	-	5
2	TiCl_4_—Ti(OBu)_4_	-	1
3	TiCl_4_—Ti(OBu)_4_	1.17	5
4	TiCl_4_—Ti(OBu)_4_	1.17	1
5	Ti(O^i^Pr)_4_—HCl	-	5
6	Ti(O^i^Pr)_4_—HCl	1.17	5
7	Ti(O^i^Pr)_4_—HCl	-	1
8	Ti(O^i^Pr)_4_—HCl	1.17	1

**Figure 1:**
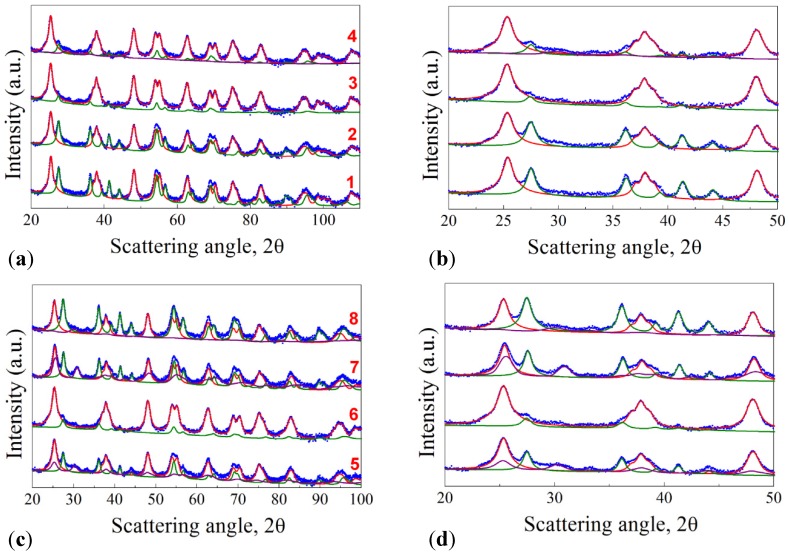
(**a**) XRPD patterns of samples 1–4; (**b**) details at low angles for samples 1–4; (**c**) XRPD patterns of samples 5–8; (**d**) details at low angles for samples 5–8.

The X-Ray Powder Diffraction (XRPD) patterns of the samples are reported in Figure 1, together with the pattern of commercial P25 annealed at 673 K. All the reported profiles were analyzed through the Rietveld method: the microstructural parameters of each phase were assigned with a high degree of accuracy, as it can be seen from the good superimposition between the green (rutile, R), pink (brookite, B), and red (anatase, A) profiles, of the Rietveld refinement, and the Bragg peaks of the experimental patterns (blue dots). As evinced in Figure 1a, samples 1–4 show the presence of both anatase and rutile phases in good agreement with previous data reported in literature [16]. From the data extrapolated by the Rietveld analysis and reported in Table 2, it is worth mentioning that the addition of acetylacetone to the initial solution induces the formation of a high content anatase-based TiO_2_ powders. If we take into account the calcination rate, the samples treated at 1 K·min^−1^ (2 and 4) show a higher rutile content, which is the thermodynamically more stable phase.

The crystallite size is almost the same for all the four samples, ranging from 18 to 20 nm for both phases and it was not influenced by the addition of coordinating ligands or by the calcination rates.

The XRD patterns of the samples 5–8 are presented in Figure 1c. Differently from the previous samples, the brookite polymorph appears when using Ti(O^i^Pr)_4_ as titanium precursor. The presence of brookite as a polymorph, together with anatase and rutile, through a sol-gel route has already been reported previously in literature [20,21]. The crystallite sizes are again in the range of 18–20 nm for both anatase and rutile, while they are more variable for brookite, ranging from 8 to 20 nm (Table 2). As already said before, no evidence of a direct influence of acetylacetone addition and of the calcination rate variation on the crystallite content can be deduced. P25 is reported for comparison and it can be seen that the A:R ratio is 83/17 while the crystallite size is much bigger than that of our samples, being 35.0 and 52.5 nm for A and R, respectively.

**Table 2:** Relative phase composition and crystallite diameters determined by Rietveld method for samples 1–8 and P25.

Samples	% anatase	% rutile	% brookite	Anatase/Rutile/Brookite average crystallite diameter (nm)
1	70	30	-	20.6/22.0
2	64	36	-	18.3/18.5
3	97	3	-	18.1/15.9
4	91	9	-	18.8/12.7
5	65	21	14	18.5/19.6/8.3
6	70	19	11	19.0/15.0/8.6
7	43	32	25	20.6/23.8/20.1
8	36	61	3	21.0/23.0/13.0
P25	83	17	-	35.0/52.5

**Figure 2:**
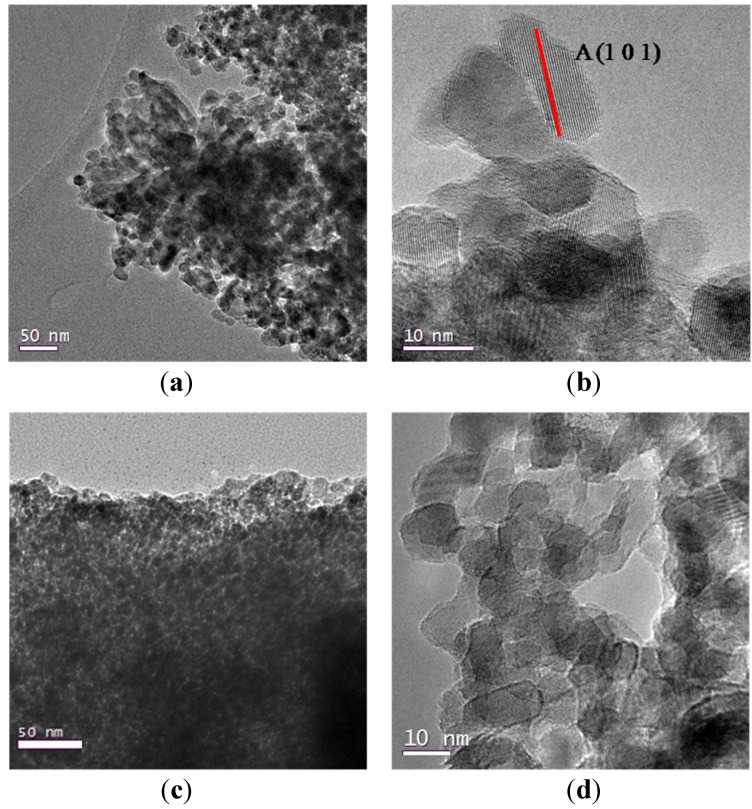
(**a**) TEM images of sample 2; (**b**) detail at high resolutions for sample 2; (**c**) TEM image of sample 3; (**d**) detail at high resolution for samples 3.

Representative TEM images of samples 2 and 3 are reported in Figure 2 and they show a highly porous structure: although they are not morphologically homogeneous, they display an appreciable order in the channels orientation in some regions of the powders, especially in sample 3 (Figure 2c); samples 1–4, synthesized from TiCl_4_ and Ti(OBu)_4_, show a similar morphology though no long range pore order can be detected; these powders possess a homogeneous particles distribution in the range of 10–30 nm. To better understand the crystalline domains, TEM images were taken also at higher resolutions (Figure 2b,d), thus allowing to appreciate the high crystallinity degree of the particles, that possess a rectangular shape associated with a truncated bipyramid with the longer axis parallel to the electron beam [22].

**Figure 3:**
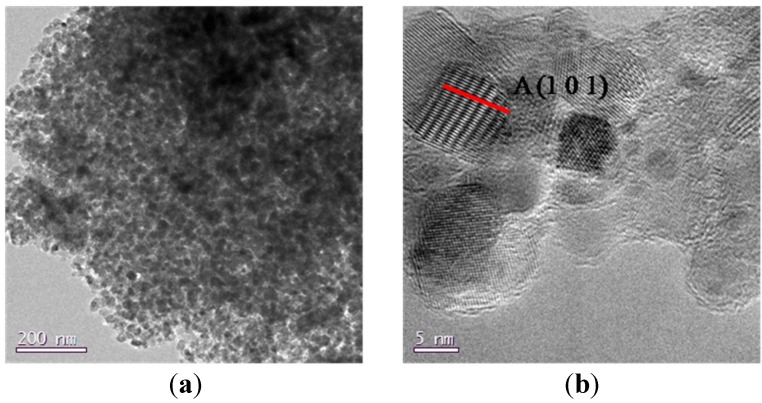
(**a**) TEM images of sample 6; (**b**) detail at high resolutions for sample 6.

Samples 5 and 6 (sample 6 is reported in Figure 3), obtained by using Ti(O^i^Pr)_4_ and HCl, both have a porous morphology and a high crystallinity degree (Figure 3b), their porous structure, displayed in TEM image of Figure 3a, shows an agglomerate of particles where it is not possible to appreciate the same channel periodicity observable in Figure 2c.

**Figure 4:**
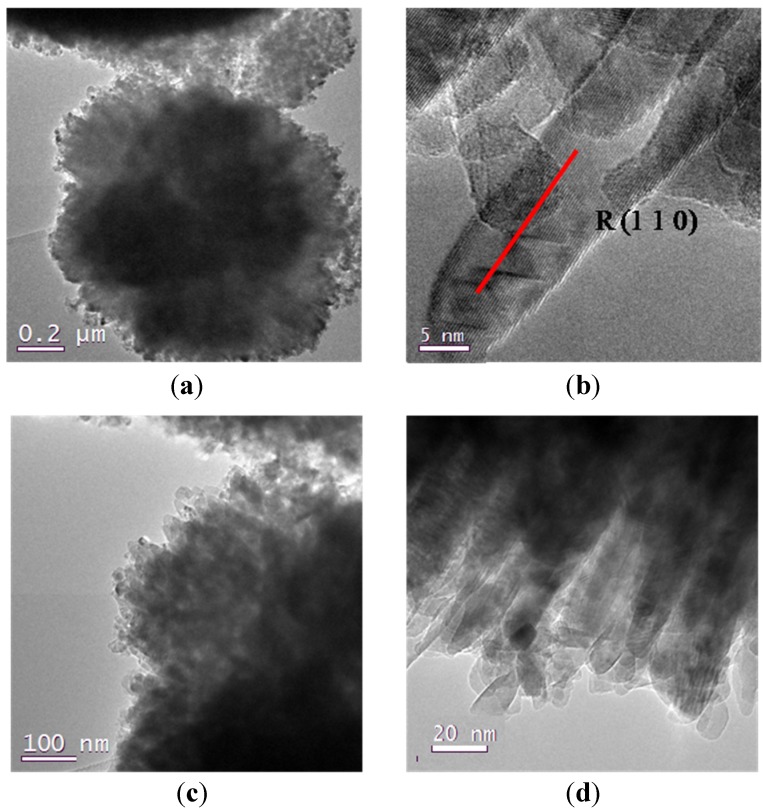
(**a**) TEM images of sample 7; (**b**) detail at high resolutions for sample 7; (**c**) TEM image of sample 8; (**d**) detail at high resolution for sample 8.

On the contrary, samples 7 and 8, also prepared from Ti(O^i^Pr)_4_ and HCl too, show a significantly different morphology of both the mesoporous framework and the crystallite shape. In Figure 4 it is clearly possible to see, as an example, the different nature of samples 7 and 8, that alternates mesoporous aggregates with much denser titania rods with a diameter of 0.5 μm, thus reducing the degree of communication between the particles. At higher resolutions, we can appreciate the difference in the crystals morphologies, characterized by a needle-like shape particles of 10–20 nm. This difference can be due to the presence of added water in the set of samples from 5 to 8, together with hydrochloric acid, that could have led to a faster hydrolysis-condensation process of the titanium precursors that did not have enough time to surround P123 micelles before starting to form titania particles.

**Figure 5:**
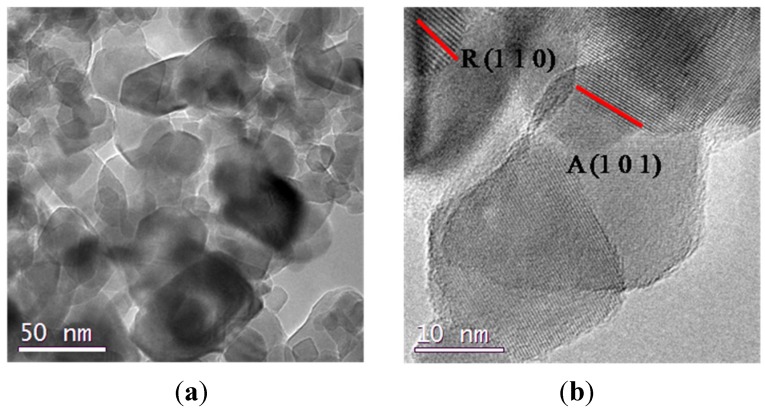
(**a**) TEM images of P25; (**b**) detail at high resolutions for P25.

P25 was analyzed by means of comparison, and in Figure 5 it is possible to see that it is composed of anatase and rutile particles, analyzed by electron diffraction, with a 20–40 nm diameter and characterized by the same rhombic and square-like morphology of our samples. The samples 1–8 possess worm-like channels (not displayed here) instead of hexagonally arranged mesopores, thus suggesting a quick gelation in which the self-assembly of P123 micelles is not completed [18].

**Figure 6:**
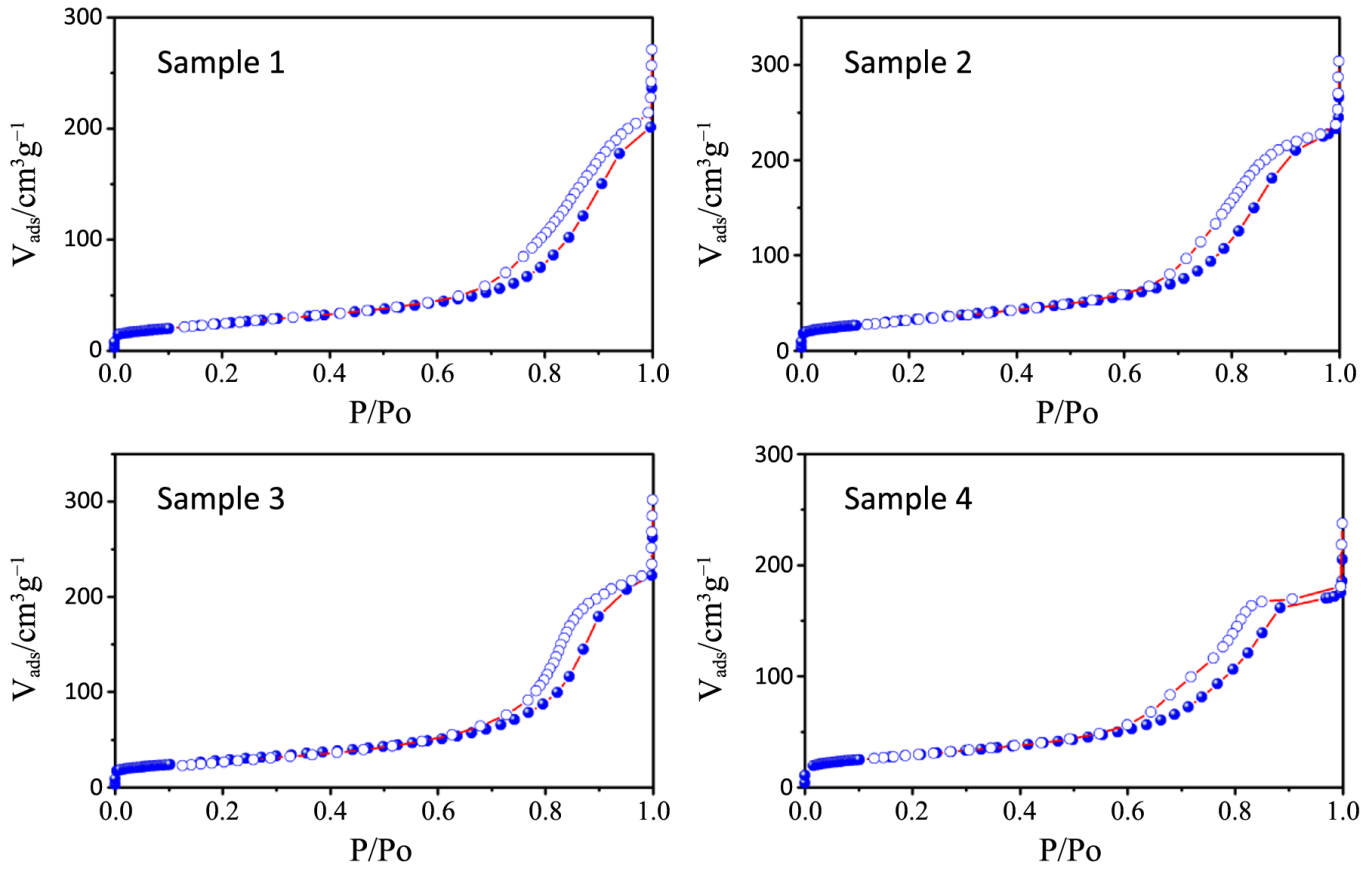
N_2_ physisorption isotherms of samples 1–4.

**Figure 7:**
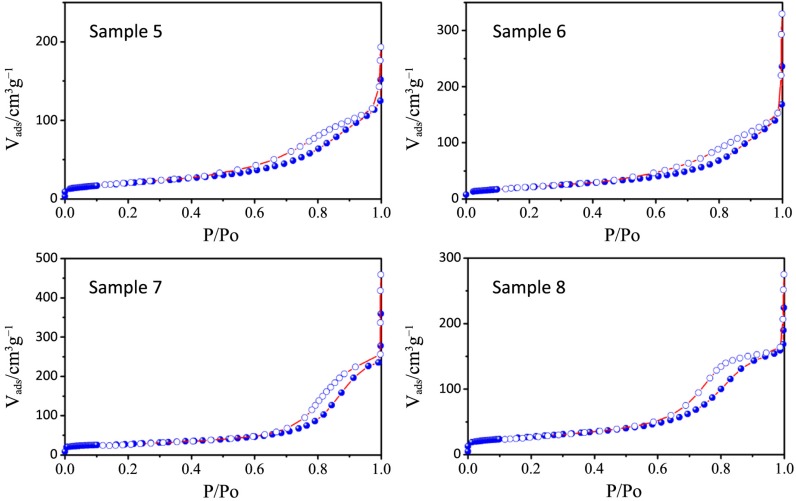
N_2_ physisorption isotherms of samples 5–8.

**Figure 8:**
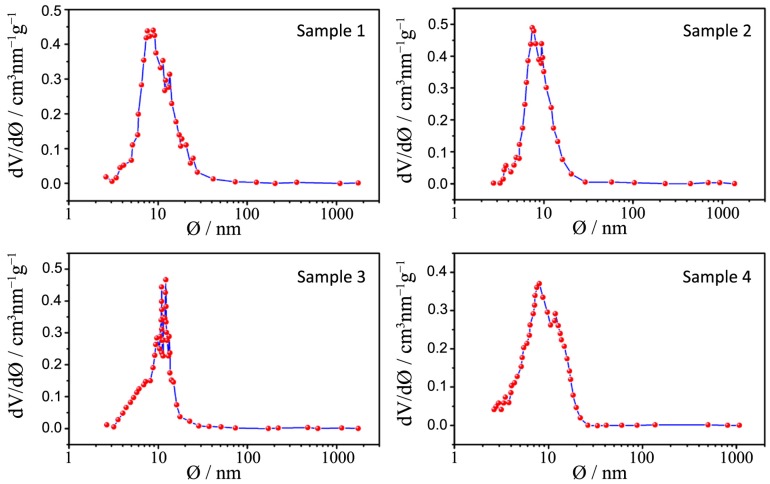
Pore size distribution plots for samples 1–4.

**Figure 9:**
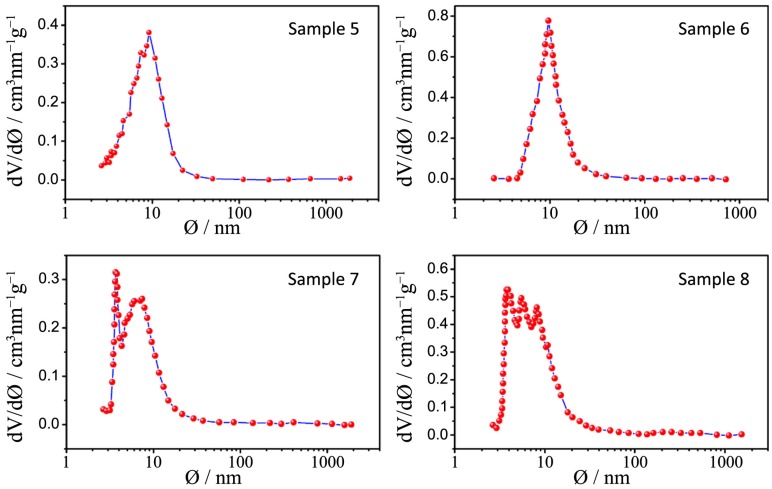
Pore size distribution plots for samples 5–8.

**Figure 10:**
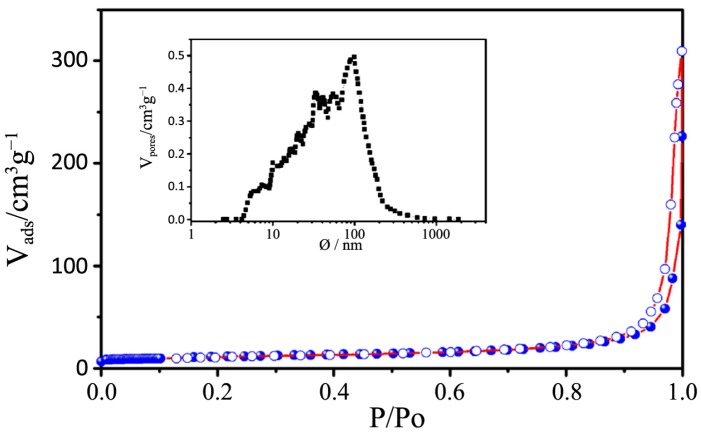
N_2_ physisorption isotherms of P25. The pore size distribution is depicted in the insert figure.

N_2_ physisorption isotherms and the pore size distribution plots of all the synthesized samples, together with that relative to P25, are depicted in Figure 6, Figure 7, Figure 8, Figure 9 and Figure 10 respectively, with the pore size distribution as an inset for P25. BET (Brunauer, Emmett, Teller) surface areas vary from 90 to 117 m^2^·g^−1^ except for samples 7 and 8, where a significant reduction to 72 and 78 m^2^·g^−1^, occurs (see Table 3). All the samples show a type IV isotherm characteristic of mesoporous materials [23] while P25 possess a type II isotherm with a H3 type hysteresis loop typical of macroporous materials. The specific surface area corresponds to 38 m^2^·g^−^^1^, with a large pore size distribution ranging from 10 to 1000 nm.

**Table 3:** BET (Brunauer, Emmett, Teller) surface area, pore volume and size for samples 1–8 and P25.

Samples	BET surface area (m^2^·g^−1^)	Pore volume (cm^3^·g^−1^)	Pore size (nm)
1	90.36	0.435	8.86
2	117.99	0.491	7.45
3	103.26	0.486	12.19
4	103.35	0.372	8.02
5	96.05	0.427	9.37
6	96.10	0.737	9.71
7	72.78	0.305	3.7
8	77.83	0.522	3.85
P25	39.00	0.474	98.03

The hysteresis loops of samples 1–4 are a H2 type while those belonging to samples 5–8 are in the middle between a H2 and a H4 type, often associated with narrow slit-shaped pores, thus suggesting a different pore structure between the two sets of samples, in accordance with TEM analysis.

All the pore size distribution plots (Figure 8 and Figure 9) present large peaks centered between 7 and 9 nm, thus in good agreement with the expected mesopore sizes when an amphiphilic triblock copolymer like Pluronic P123 is used (in the range of several nanometers to 10 nm) [24]. However, some exceptions can be underlined: a shift toward smaller diameters can be appreciated for samples 7 and 8 (around 3 nm) and this is probably due, as already said, to both a quick hydrolysis-condensation process during gelation and to a subsequent collapse of the partially formed mesostructure during calcination [18,25].

The large distribution of the pore sizes has probably two different causes: the first one, as already mentioned, is the incomplete formation of the mesostructure that lead to a disordered mesoporous morphology and the second is the presence of both inter-particle pores and assembled pores [26]. As a matter of fact, in the synthesized powders it is possible to find two different types of pores, one is the interparticle pores formed from the packing of neighboring particles and the other is the assembled pores that represent the position previously occupied by the surfactant P123.

A really ordered mesostructure is obtained only when interparticle and assembled pores are of the same size [26] and in our powders this aim is not achieved because of the previously illustrated issues. However it is worth mentioning the distribution of sample 3: in this case the peaks are more defined and narrow and are centered at around 12 nm, which is a higher value with respect to other samples; in this sample interparticles and assembled pores are closer in size and this can lead to a higher degree of communication between particles and to a more ordered structure.

All the powders, P25 included, were then used to fabricate photoanodes for water splitting experiments with the procedure reported in the experimental section.

**Figure 11:**
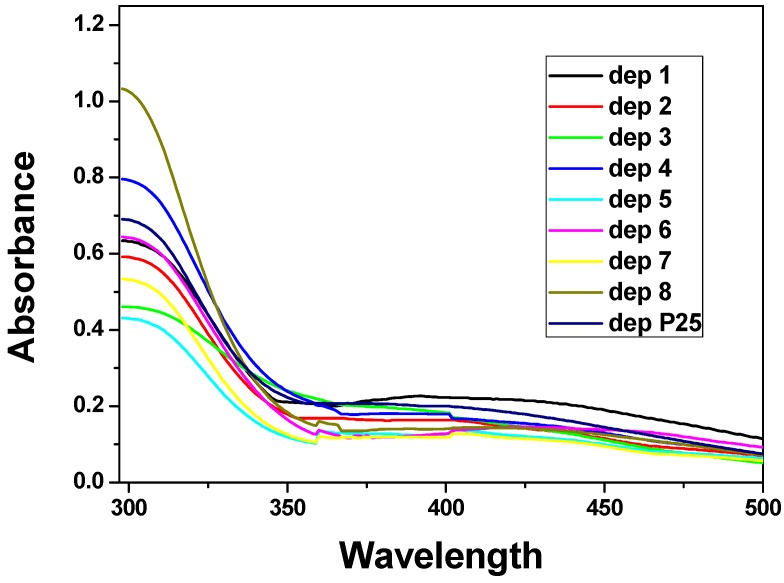
UV-Vis spectra (electronic spectra in the ultraviolet and visible region) of samples 1–8 and P25.

UV-Vis absorption spectra were recorded on the electrodes and the response can be seen in Figure 11.

In all the spectra a strong sharp increase in absorption appears at about 360 nm and ends with a peak at about 300 nm, corresponding to the titania semiconductor. The band gap values (Table 4) were calculated from the onset of the wavelength (λ_onset_) identified by the tangent method. The band gap varies from 3.18 to 3.55 eV. These values are higher than those reported in literature for bulk titania (3.02 eV for rutile and 3.20 eV for anatase) [27] and this is attributed to the small size of our titania nanocrystallites (quantum-size effect) [28]. The broad absorption bands in the visible region (550–400 nm) are attributable to interference colors resulting from the thickness of the films [29].

**Table 4:** λ_onset_ and band gap from UV-Vis (UltraViolet-Visible) spectra of samples 1–8 and P25.

Samples	λ_onset_ (nm)	Band gap (eV)
1	362	3.41
2	363	3.42
3	388	3.18
4	359	3.44
5	351	3.53
6	361	3.43
7	354	3.47
8	349	3.55
P25	361	3.44

**Figure 12:**
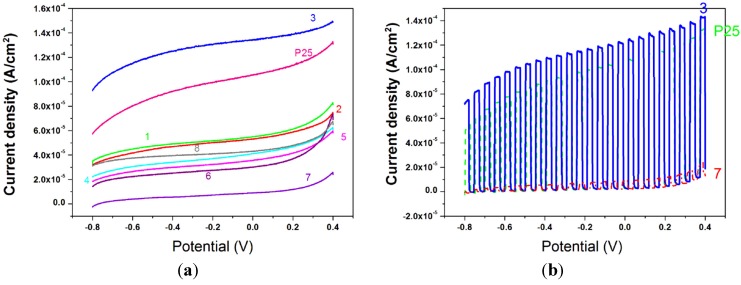
(**a**) J-V curves under illumination for samples 1–8 and P25. WE = TiO_2_/ITO, RE = Ag/AgCl, CE = Pt, NaOH 1M, 5 mV·s^−1^; (**b**) J-V curves under chopped light for samples 3, 7, and P25. WE = TiO_2_/ITO, RE = Ag/AgCl, CE = Pt, NaOH 1 M, 5 mV·s^−1^.

The films were then tested as photoelectrodes for the water splitting reaction. Linear Sweep Voltammetry was used to record (photo)current density-applied voltage responses in a 1 M NaOH solution at a scan rate of 10 mV·s^−1^. The ending potential of the measurements, both in the dark and under 1 sun illumination power (AM1.5G), was chosen, from previous scans in the dark, to avoid electrochemical oxidation of water due to a raising concentration of holes in titania valence band. The curves registered under illumination are presented in Figure 12a, the curves registered under chopped light for sample 3, 7, and for P25, are reported in Figure 12b and the photocurrent densities at 0 V bias *vs*. Ag/AgCl are in Table 5. P25, one of the most photoactive titania powders so far, gives a current of 105 μA·cm^−2^. Almost all the samples give responses from 30 to 55 μA·cm^−2^ but two exceptions can be appreciated. Sample 7, synthesized from Ti(O^i^Pr)_4_ and HCl without the addition of acetylacetone, is the one that gives far the worst result in photoactivity while sample 3 (TiCl_4_ Ti(OBu)_4_ and acetylacetone) demonstrate to be even better than P25 with a photocurrent value of 134 μA·cm^−2^. By analyzing the differences between these two samples, 3 and 7, we saw that sample 3 is the one with the highest amount of anatase (Table 2), which is known to be the most photoactive titania polymorph, while sample 7 is not the one with the lowest anatase content, but it reveals the highest amount of brookite which probably reasons the low photoactivity of the sample itself. Although the role of brookite in titania photoactivity has never been deeply studied, some authors found that, together with anatase, brookite leads to high photocatalytic performances in *n*-pentane oxidation [30], but in our case the contemporary presence of rutile may play an important role by establishing interactions with the two other phases that are not fully clarified yet. Focusing on the surface area (Table 3), sample 3 has a high area but not the highest one, which was of sample 2. The pore volume (Table 3) is not negligible with 0.485 cm^3^·g^−1^ but is quite low in respect to sample 6, 0.736 cm^3^·g^−1^ or even to sample 7 (0.522 cm^3^·g^−1^). The main difference lays in the much narrower pore size distribution (inset Figure 3) and the bigger pore diameters (12 nm). All these considerations lead to the conclusion that not only a high content of brookite may worsen the photocatalytic activity, but also that neither the pore volume nor the surface area play a key role in the electrodes performances; the most important parameter seems to be the homogeneous intra- and inter-particles pore distribution, which probably leads to an optimized mass transfer and, as a consequence, to a better photoactivity. Further experiments are planned in order to study the mass transport in these powders (e.g., Impedance Spectroscopy).

**Table 5:** Current densities at 0 V *vs.* Ag/AgCl for samples 1–8 and P25.

Samples	J (µA·cm^2^)	Volts
1	55	0
2	53	0
3	134	0
4	41	0
5	36	0
6	30	0
7	9	0
8	43	0
P25	105	0

The electrodes were then tested by exposing them, in the electrochemical cell, to a chopped light under 0 V bias for an hour while monitoring the photocurrent profile. All the electrodes lost no more than 5% of their initial current, showing good mechanical resistance between the powders and the conductive substrate (ITO) and good chemical stability, all precious characteristics for a future application.

## 3. Experimental Section

Commercial TiO_2_ powders (>99.5%) were purchased from Sigma-Aldrich, Sigma-Aldrich Corporation, St. Louis, Missouri, USA. Synthetic procedures were a modification of some already reported in literature [16]. In a typical synthesis, 1 g of surfactant (Pluronic P123 from Sigma Aldrich) was dissolved in 20 mL of ethanol, then in some cases 1.17 mL of acetylacetone were added, subsequently 1 mL of TiCl_4_ and 0.78 mL of Ti(OBu)_4_ or 2.94 mL of Ti(O^i^Pr)_4_ with 3 mL of HCl 12M are put in the previous solution. The mixture was kept under stirring for two hours at room temperature and is then transferred in a Petri dish and put at 318 K with a moisture level, measured with an hygrometer, of 40%–60% for 24 h. The obtained gel was finally calcined at 673 K for 12 h using different rates, 1 K·min^−1^ or 5 K·min^−1^. All the syntheses are reported in Table 1.

The preparation of the electrodes for photoelectrochemical measurements started with creating a paste with 20 mg of each sample, 15 mg of polyethylene glycol (PEG 20,000 g/mol) and 1.6 mL of ethanol. The paste was then sonicated until homogeneous and is then deposited onto an indium tin oxide (ITO) glass with a conductive area of 1 cm^2^ by the doctor blade technique using four pieces of adhesive tape to control the thickness of the titania layer. The TiO_2_/ITO deposites were then thermally treated in a muffle furnace at 673 K for one hour.

The structural characterization of the samples was carried out by XRPD (Rigaku DMax diffractometer, Rigaku Corporation, Tokyo, Japan, equipped with a graphite monochromator on the diffracted beam), and Transmission Electron Microscopy TEM (Jeol-JEM 2011, 200 kW, Jeol Ltd., Tokyo, Japan).

For TEM observations, samples were prepared by dispersing a few milligrams in ethanol followed by the deposition of one or two drops of the suspension on a holey carbon supported grid.

The Rietveld method through Maud software [31] was used for qualitative and quantitative analysis of phase content and microstructural parameters.

N_2_ sorption isotherms were collected with a Sorptomatic 1990 instrument (Fisons Instruments, Milan, Italy); the superficial area was calculated with BET method while the pore distribution with BJH method. 200 mg of each sample were put in a quartz tube and degassed under vacuum (1 × 10^−3^ bar) at 523 K for 24 h. The dead volume was evaluated through helium measurements.

UV-Vis spectra were recorded with a Hitachi U-2010 (Hitachi Ltd, Tokyo, Japan) directly on the titania electrodes.

Photoelectrochemical characterizations were performed in a teflon lined quartz cell (PECC-1 from Zhaner, Kansas City, Missouri, USA) under inert atmosphere in a degassed 1 M NaOH solution using the prepared TiO_2_/ITO electrodes as working electrodes, a Pt wire as counter electrode and Ag/AgCl as a reference electrode (E = 207.6 mV *vs.* NHE). Linear Sweep Voltammetry (LSV) responses were recorded at 10 mV·s^−1^. Experiments were run starting from the open circuit potential of the system, first in the dark and then under direct illumination, under the control of a Zhaner Zennium Mess Systeme PP221 potentiostat, Zahner, Kansas City, MI, USA. The light source was a LOT Oriel Solar Simulator (LOT- QuantumDesign, Darmstadt, Germany) with AM (air mass) 1.5 G filter, ozone free and with an output power of 1 sun. The polarization experiments were carried out with chopped light at 0 V *vs.* Ag/AgCl for an hour in the same electrochemical system.

## 4. Conclusions

Mesoporous titania powders with a nanocrystalline framework were successfully synthesized through a simple EISA approach. The influence of different titania precursors, calcination rates and ligand addition on titania phase content and morphology was analyzed, concluding that the acid base approach lead to rutile-anatase mixed phases with an anatase predominance in conjunction with acetylacetone addition, while in the presence of Ti(O^i^Pr)_4_ and HCl the brookite polymorph appeared. The samples, all compared to commercial P25, showed a mesoporous structure though no long range order could be detected. Surface areas were in the range between 72 and 117 m^2^·g^−1^, all higher than P25 area of 38 m^2^·g^−1^ the porous framework seemed to get worse with the use of HCl and the powders showed dense agglomerates alternated to porous portions, thus leading to an appreciable decrease of the surface areas. The calcination rate was found to be of no influence on both the crystalline and mesoporous domains. All powders were tested as photoanodes for the water splitting reaction and the results were compared to commercial P25. Among them, the sample with a narrower pore size distribution and a higher anatase content possessed notable photoactivity, even higher than P25, leading to the conclusion that the most important parameter is the presence of a high degree of communication between the particles, that is guaranteed by similar pore sizes.
